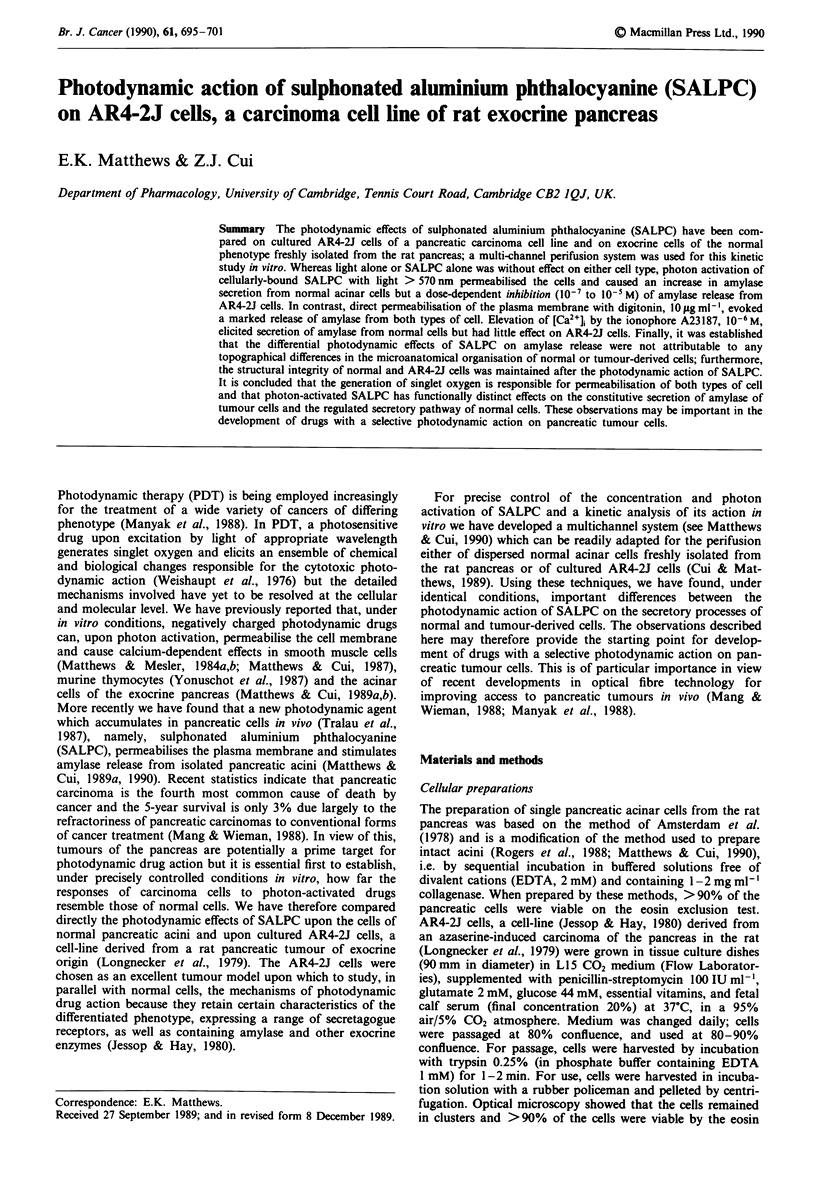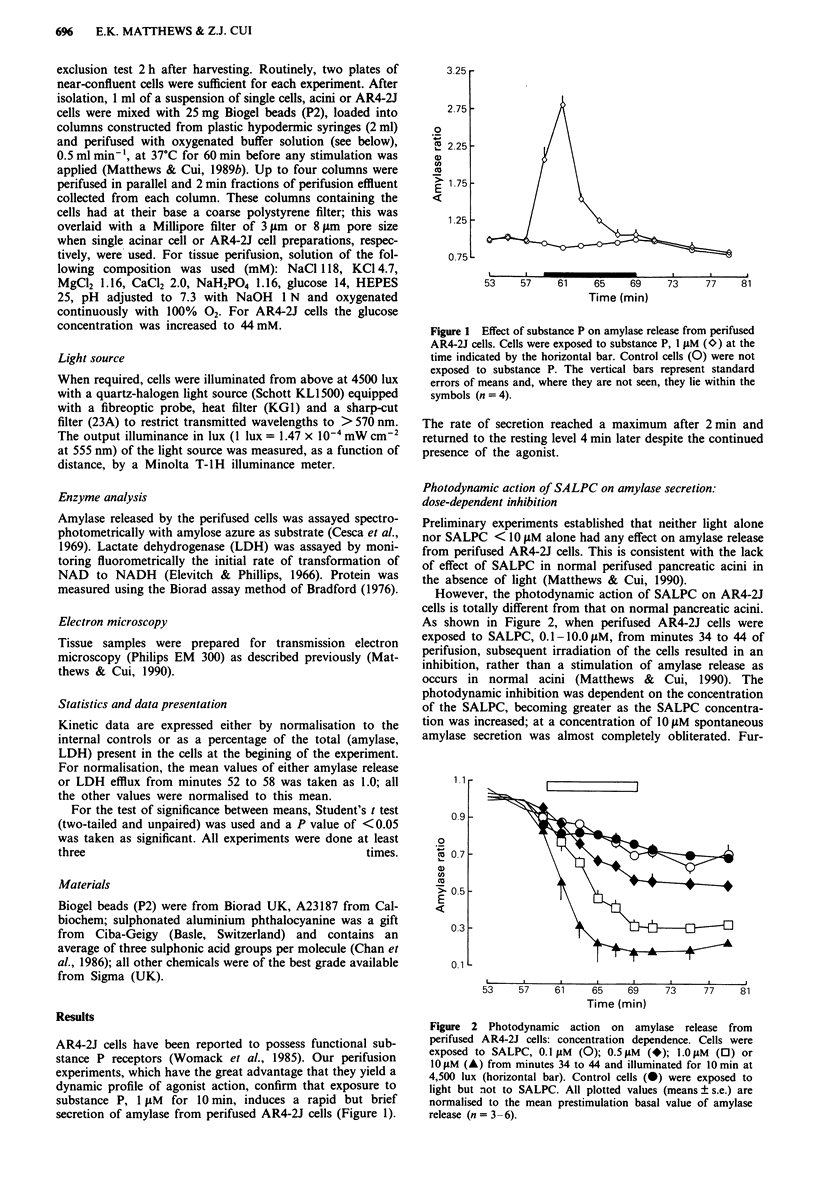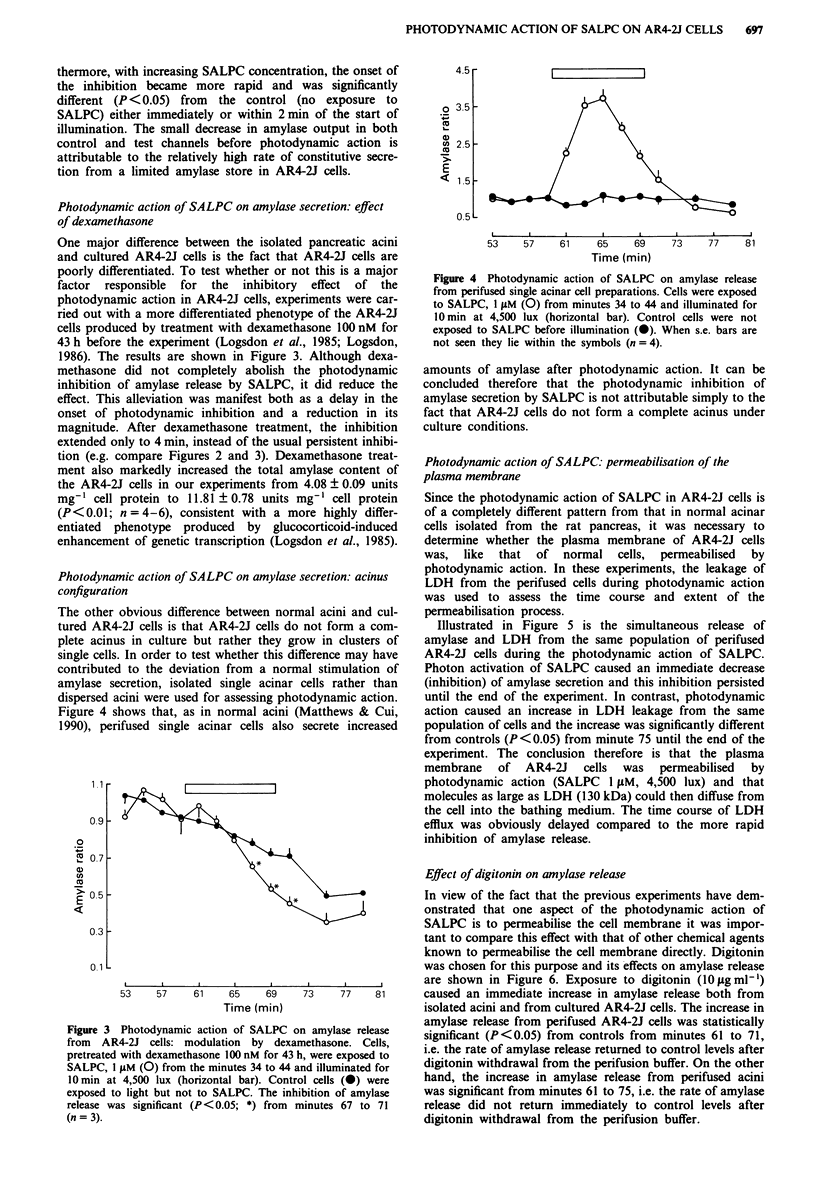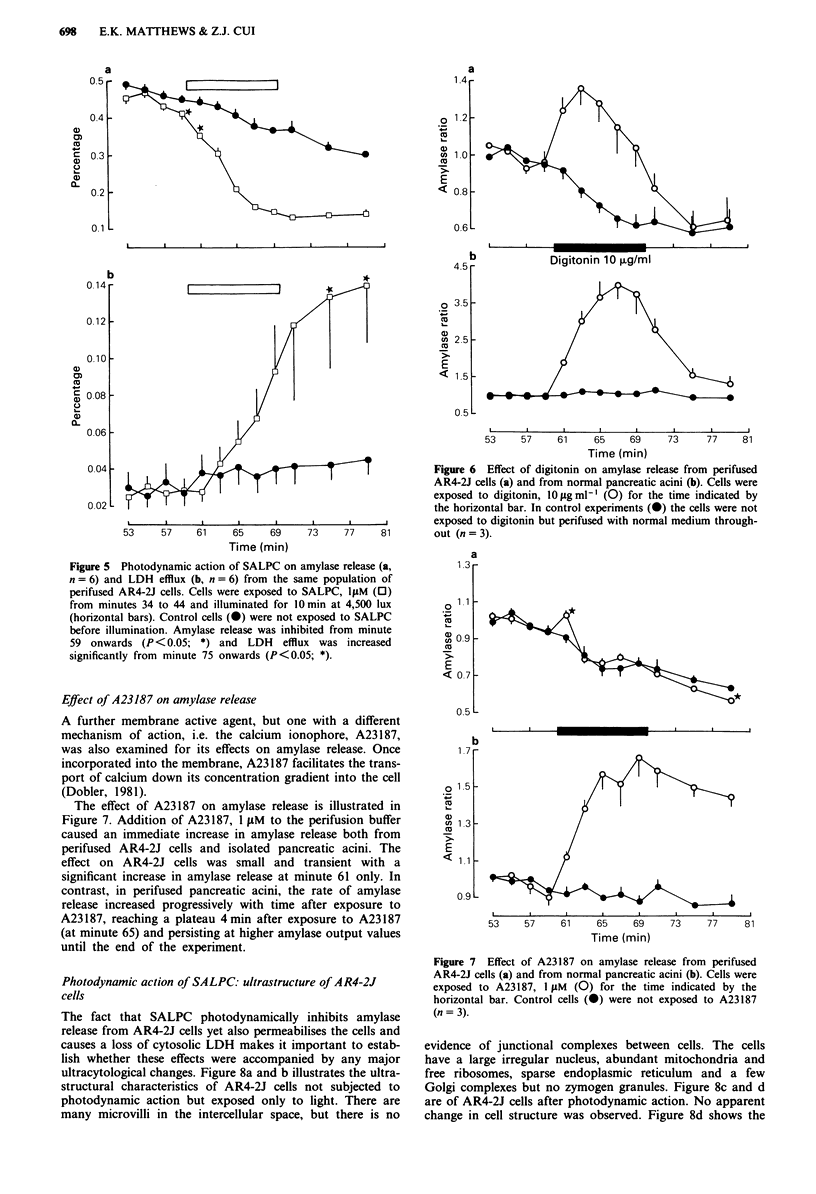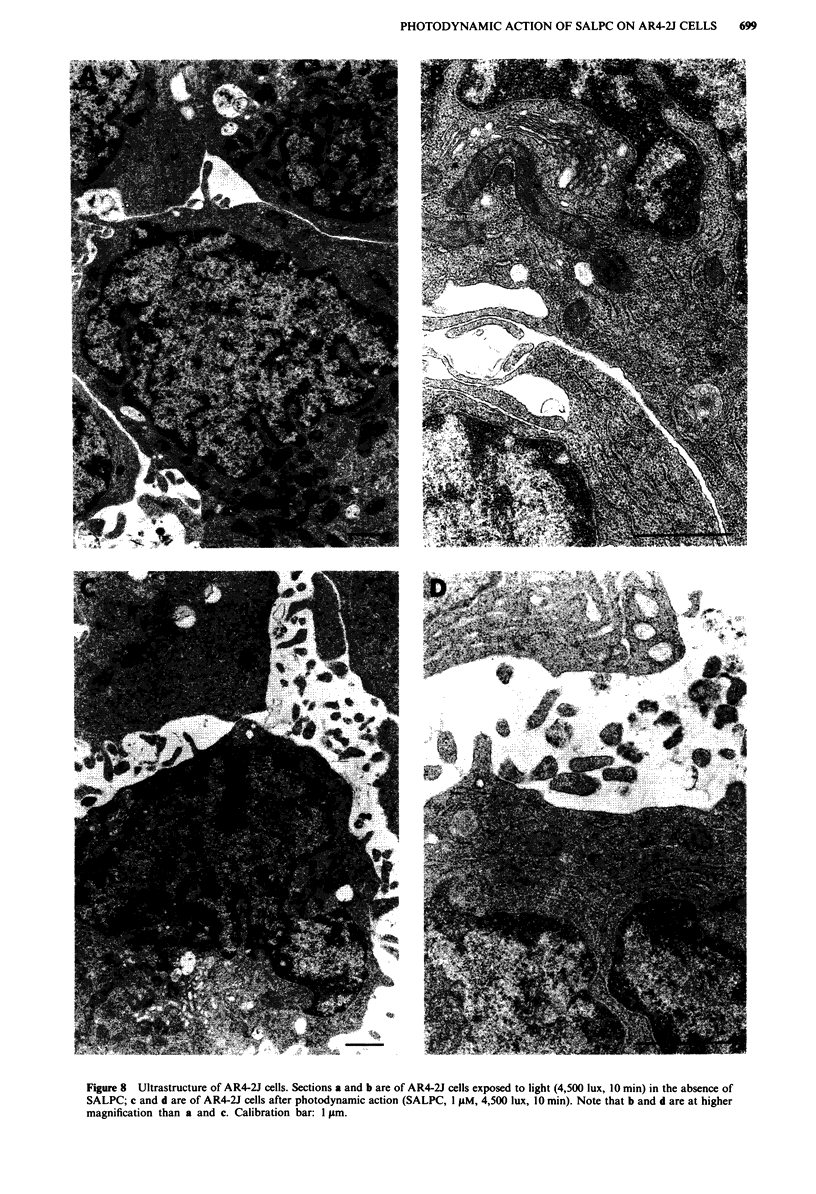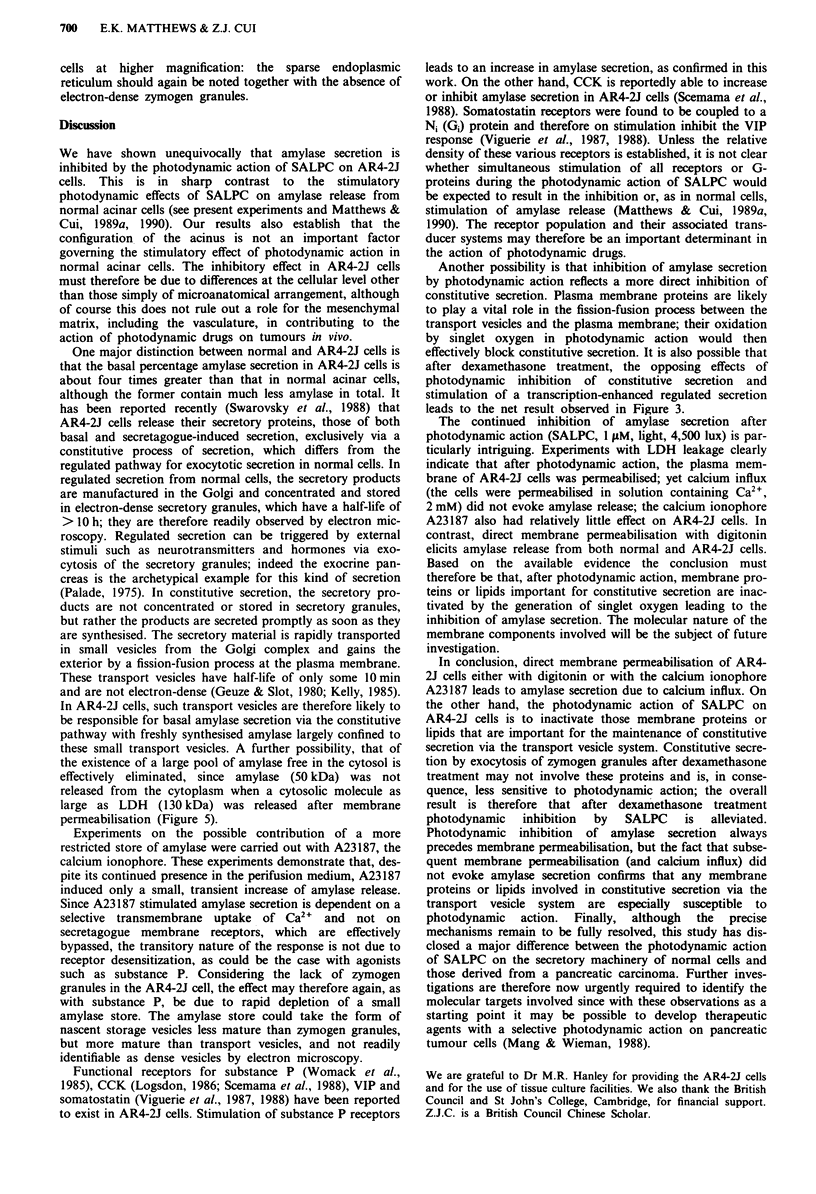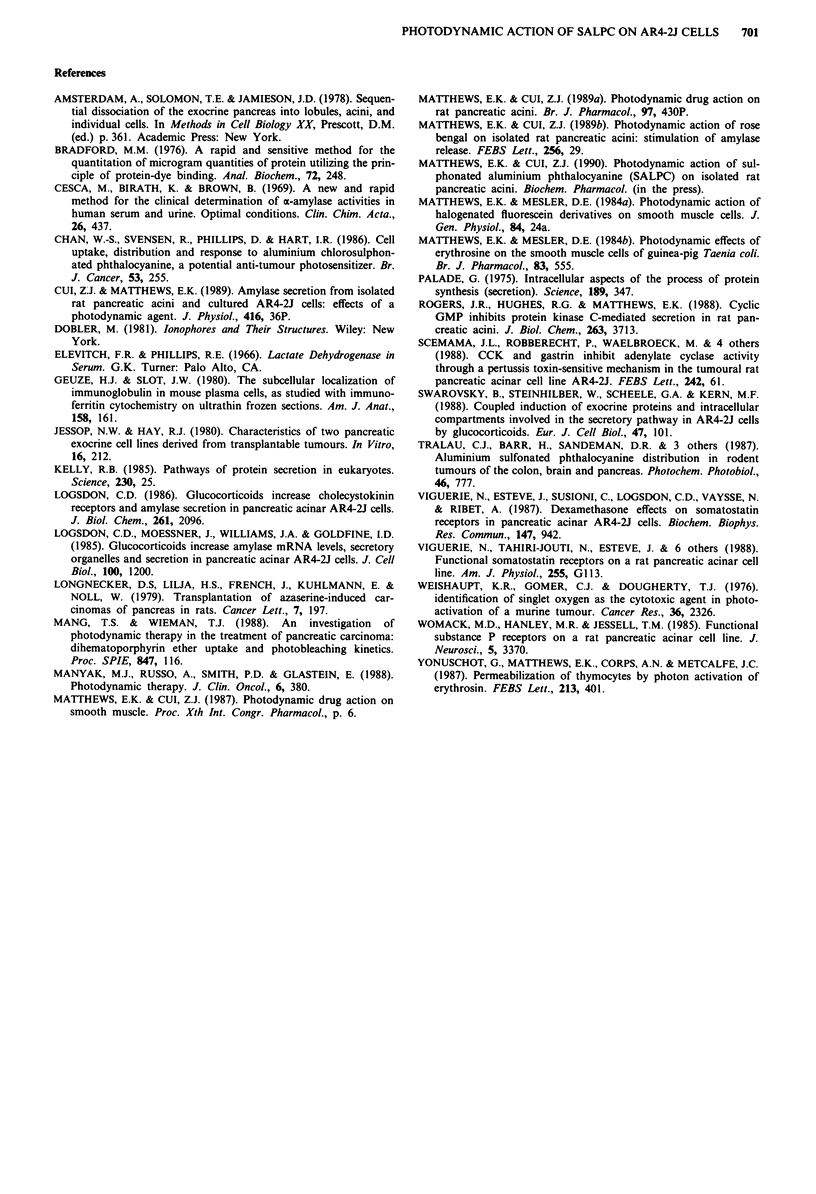# Photodynamic action of sulphonated aluminium phthalocyanine (SALPC) on AR4-2J cells, a carcinoma cell line of rat exocrine pancreas.

**DOI:** 10.1038/bjc.1990.157

**Published:** 1990-05

**Authors:** E. K. Matthews, Z. J. Cui

**Affiliations:** Department of Pharmacology, University of Cambridge, UK.

## Abstract

**Images:**


Br. J. Cancer (1990), 61, 695–701 © Macmillan Press Ltd., 1990

### Photodynamic action of sulphonated aluminium phthalocyanine (SALPC) on AR4-2J cells, a carcinoma cell line of rat exocrine pancreas

E.K. Matthews & Z.J. Cui

*Department of Pharmacology, University of Cambridge, Tennis Court Road, Cambridge CB2 1QJ, UK.*

**Summary** The photodynamic effects of sulphonated aluminium phthalocyanine (SALPC) have been compared on cultured AR4-2J cells of a pancreatic carcinoma cell line and on exocrine cells of the normal phenotype freshly isolated from the rat pancreas; a multi-channel perifusion system was used for this kinetic study *in vitro*. Whereas light alone or SALPC alone was without effect on either cell type, photon activation of cellularly-bound SALPC with light > 570 nm permeabilised the cells and caused an increase in amylase secretion from normal acinar cells but a dose-dependent *inhibition* ($10^{-7}$ to $10^{-5}$ M) of amylase release from AR4-2J cells. In contrast, direct permeabilisation of the plasma membrane with digitonin, 10 $\mu g\ ml^{-1}$, evoked a marked release of amylase from both types of cell. Elevation of $[Ca^{2+}]_i$ by the ionophore A23187, $10^{-6}$ M, elicited secretion of amylase from normal cells but had little effect on AR4-2J cells. Finally, it was established that the differential photodynamic effects of SALPC on amylase release were not attributable to any topographical differences in the microanatomical organisation of normal or tumour-derived cells; furthermore, the structural integrity of normal and AR4-2J cells was maintained after the photodynamic action of SALPC. It is concluded that the generation of singlet oxygen is responsible for permeabilisation of both types of cell and that photon-activated SALPC has functionally distinct effects on the constitutive secretion of amylase of tumour cells and the regulated secretory pathway of normal cells. These observations may be important in the development of drugs with a selective photodynamic action on pancreatic tumour cells.

Photodynamic therapy (PDT) is being employed increasingly for the treatment of a wide variety of cancers of differing phenotype (Manyak *et al.*, 1988). In PDT, a photosensitive drug upon excitation by light of appropriate wavelength generates singlet oxygen and elicits an ensemble of chemical and biological changes responsible for the cytotoxic photodynamic action (Weishaupt *et al.*, 1976) but the detailed mechanisms involved have yet to be resolved at the cellular and molecular level. We have previously reported that, under *in vitro* conditions, negatively charged photodynamic drugs can, upon photon activation, permeabilise the cell membrane and cause calcium-dependent effects in smooth muscle cells (Matthews & Mesler, 1984*a,b*; Matthews & Cui, 1987), murine thymocytes (Yonuschot *et al.*, 1987) and the acinar cells of the exocrine pancreas (Matthews & Cui, 1989*a,b*). More recently we have found that a new photodynamic agent which accumulates in pancreatic cells *in vivo* (Tralau *et al.*, 1987), namely, sulphonated aluminium phthalocyanine (SALPC), permeabilises the plasma membrane and stimulates amylase release from isolated pancreatic acini (Matthews & Cui, 1989*a*, 1990). Recent statistics indicate that pancreatic carcinoma is the fourth most common cause of death by cancer and the 5-year survival is only 3% due largely to the refractoriness of pancreatic carcinomas to conventional forms of cancer treatment (Mang & Wieman, 1988). In view of this, tumours of the pancreas are potentially a prime target for photodynamic drug action but it is essential first to establish, under precisely controlled conditions *in vitro*, how far the responses of carcinoma cells to photon-activated drugs resemble those of normal cells. We have therefore compared directly the photodynamic effects of SALPC upon the cells of normal pancreatic acini and upon cultured AR4-2J cells, a cell-line derived from a rat pancreatic tumour of exocrine origin (Longnecker *et al.*, 1979). The AR4-2J cells were chosen as an excellent tumour model upon which to study, in parallel with normal cells, the mechanisms of photodynamic drug action because they retain certain characteristics of the differentiated phenotype, expressing a range of secretagogue receptors, as well as containing amylase and other exocrine enzymes (Jessop & Hay, 1980).

For precise control of the concentration and photon activation of SALPC and a kinetic analysis of its action *in vitro* we have developed a multichannel system (see Matthews & Cui, 1990) which can be readily adapted for the perifusion either of dispersed normal acinar cells freshly isolated from the rat pancreas or of cultured AR4-2J cells (Cui & Matthews, 1989). Using these techniques, we have found, under identical conditions, important differences between the photodynamic action of SALPC on the secretory processes of normal and tumour-derived cells. The observations described here may therefore provide the starting point for development of drugs with a selective photodynamic action on pancreatic tumour cells. This is of particular importance in view of recent developments in optical fibre technology for improving access to pancreatic tumours *in vivo* (Mang & Wieman, 1988; Manyak *et al.*, 1988).

### Materials and methods

### Cellular preparations

The preparation of single pancreatic acinar cells from the rat pancreas was based on the method of Amsterdam *et al.* (1978) and is a modification of the method used to prepare intact acini (Rogers *et al.*, 1988; Matthews & Cui, 1990), i.e. by sequential incubation in buffered solutions free of divalent cations (EDTA, 2 mM) and containing 1–2 $mg\ ml^{-1}$ collagenase. When prepared by these methods, >90% of the pancreatic cells were viable on the eosin exclusion test. AR4-2J cells, a cell-line (Jessop & Hay, 1980) derived from an azaserine-induced carcinoma of the pancreas in the rat (Longnecker *et al.*, 1979) were grown in tissue culture dishes (90 mm in diameter) in L15 $CO_2$ medium (Flow Laboratories), supplemented with penicillin-streptomycin 100 $IU\ ml^{-1}$, glutamate 2 mM, glucose 44 mM, essential vitamins, and fetal calf serum (final concentration 20%) at 37°C, in a 95% air/5% $CO_2$ atmosphere. Medium was changed daily; cells were passaged at 80% confluence, and used at 80–90% confluence. For passage, cells were harvested by incubation with trypsin 0.25% (in phosphate buffer containing EDTA 1 mM) for 1–2 min. For use, cells were harvested in incubation solution with a rubber policeman and pelleted by centrifugation. Optical microscopy showed that the cells remained in clusters and >90% of the cells were viable by the eosin

Correspondence: E.K. Matthews.

Received 27 September 1989; and in revised form 8 December 1989.

exclusion test 2 h after harvesting. Routinely, two plates of near-confluent cells were sufficient for each experiment. After isolation, 1 ml of a suspension of single cells, acini or AR4-2J cells were mixed with 25 mg Biogel beads (P2), loaded into columns constructed from plastic hypodermic syringes (2 ml) and perifused with oxygenated buffer solution (see below), 0.5 ml $min^{-1}$, at 37°C for 60 min before any stimulation was applied (Matthews & Cui, 1989*b*). Up to four columns were perifused in parallel and 2 min fractions of perifusion effluent collected from each column. These columns containing the cells had at their base a coarse polystyrene filter; this was overlaid with a Millipore filter of 3 μm or 8 μm pore size when single acinar cell or AR4-2J cell preparations, respectively, were used. For tissue perifusion, solution of the following composition was used (mM): NaCl 118, KCl 4.7, $MgCl_2$ 1.16, $CaCl_2$ 2.0, $NaH_2PO_4$ 1.16, glucose 14, HEPES 25, pH adjusted to 7.3 with NaOH 1 N and oxygenated continuously with 100% $O_2$. For AR4-2J cells the glucose concentration was increased to 44 mM.

### *Light source*

When required, cells were illuminated from above at 4500 lux with a quartz-halogen light source (Schott KL1500) equipped with a fibreoptic probe, heat filter (KG1) and a sharp-cut filter (23A) to restrict transmitted wavelengths to > 570 nm. The output illuminance in lux (1 lux = $1.47 \times 10^{-4}$ mW $cm^{-2}$ at 555 nm) of the light source was measured, as a function of distance, by a Minolta T-1H illuminance meter.

### *Enzyme analysis*

Amylase released by the perifused cells was assayed spectrophotometrically with amylose azure as substrate (Cesca *et al.*, 1969). Lactate dehydrogenase (LDH) was assayed by monitoring fluorometrically the initial rate of transformation of NAD to NADH (Elevitch & Phillips, 1966). Protein was measured using the Biorad assay method of Bradford (1976).

### *Electron microscopy*

Tissue samples were prepared for transmission electron microscopy (Philips EM 300) as described previously (Matthews & Cui, 1990).

### *Statistics and data presentation*

Kinetic data are expressed either by normalisation to the internal controls or as a percentage of the total (amylase, LDH) present in the cells at the begining of the experiment. For normalisation, the mean values of either amylase release or LDH efflux from minutes 52 to 58 was taken as 1.0; all the other values were normalised to this mean.

For the test of significance between means, Student's *t* test (two-tailed and unpaired) was used and a *P* value of < 0.05 was taken as significant. All experiments were done at least three times.

### *Materials*

Biogel beads (P2) were from Biorad UK, A23187 from Calbiochem; sulphonated aluminium phthalocyanine was a gift from Ciba-Geigy (Basle, Switzerland) and contains an average of three sulphonic acid groups per molecule (Chan *et al.*, 1986); all other chemicals were of the best grade available from Sigma (UK).

### Results

AR4-2J cells have been reported to possess functional substance P receptors (Womack *et al.*, 1985). Our perifusion experiments, which have the great advantage that they yield a dynamic profile of agonist action, confirm that exposure to substance P, 1 μM for 10 min, induces a rapid but brief secretion of amylase from perifused AR4-2J cells (Figure 1).

**Figure 1** Effect of substance P on amylase release from perifused AR4-2J cells. Cells were exposed to substance P, 1 μM (◇) at the time indicated by the horizontal bar. Control cells (○) were not exposed to substance P. The vertical bars represent standard errors of means and, where they are not seen, they lie within the symbols ($n = 4$).

The rate of secretion reached a maximum after 2 min and returned to the resting level 4 min later despite the continued presence of the agonist.

### *Photodynamic action of SALPC on amylase secretion: dose-dependent inhibition*

Preliminary experiments established that neither light alone nor SALPC < 10 μM alone had any effect on amylase release from perifused AR4-2J cells. This is consistent with the lack of effect of SALPC in normal perifused pancreatic acini in the absence of light (Matthews & Cui, 1990).

However, the photodynamic action of SALPC on AR4-2J cells is totally different from that on normal pancreatic acini. As shown in Figure 2, when perifused AR4-2J cells were exposed to SALPC, 0.1 – 10.0 μM, from minutes 34 to 44 of perifusion, subsequent irradiation of the cells resulted in an inhibition, rather than a stimulation of amylase release as occurs in normal acini (Matthews & Cui, 1990). The photodynamic inhibition was dependent on the concentration of the SALPC, becoming greater as the SALPC concentration was increased; at a concentration of 10 μM spontaneous amylase secretion was almost completely obliterated. Fur-

**Figure 2** Photodynamic action on amylase release from perifused AR4-2J cells: concentration dependence. Cells were exposed to SALPC, 0.1 μM (○); 0.5 μM (◆); 1.0 μM (□) or 10 μM (▲) from minutes 34 to 44 and illuminated for 10 min at 4,500 lux (horizontal bar). Control cells (●) were exposed to light but not to SALPC. All plotted values (means ± s.e.) are normalised to the mean prestimulation basal value of amylase release ($n = 3–6$).

thermore, with increasing SALPC concentration, the onset of the inhibition became more rapid and was significantly different ($P<0.05$) from the control (no exposure to SALPC) either immediately or within 2 min of the start of illumination. The small decrease in amylase output in both control and test channels before photodynamic action is attributable to the relatively high rate of constitutive secretion from a limited amylase store in AR4-2J cells.

### *Photodynamic action of SALPC on amylase secretion: effect of dexamethasone*

One major difference between the isolated pancreatic acini and cultured AR4-2J cells is the fact that AR4-2J cells are poorly differentiated. To test whether or not this is a major factor responsible for the inhibitory effect of the photodynamic action in AR4-2J cells, experiments were carried out with a more differentiated phenotype of the AR4-2J cells produced by treatment with dexamethasone 100 nM for 43 h before the experiment (Logsdon *et al.*, 1985; Logsdon, 1986). The results are shown in Figure 3. Although dexamethasone did not completely abolish the photodynamic inhibition of amylase release by SALPC, it did reduce the effect. This alleviation was manifest both as a delay in the onset of photodynamic inhibition and a reduction in its magnitude. After dexamethasone treatment, the inhibition extended only to 4 min, instead of the usual persistent inhibition (e.g. compare Figures 2 and 3). Dexamethasone treatment also markedly increased the total amylase content of the AR4-2J cells in our experiments from 4.08 ± 0.09 units $mg^{-1}$ cell protein to 11.81 ± 0.78 units $mg^{-1}$ cell protein ($P<0.01$; $n = 4-6$), consistent with a more highly differentiated phenotype produced by glucocorticoid-induced enhancement of genetic transcription (Logsdon *et al.*, 1985).

### *Photodynamic action of SALPC on amylase secretion: acinus configuration*

The other obvious difference between normal acini and cultured AR4-2J cells is that AR4-2J cells do not form a complete acinus in culture but rather they grow in clusters of single cells. In order to test whether this difference may have contributed to the deviation from a normal stimulation of amylase secretion, isolated single acinar cells rather than dispersed acini were used for assessing photodynamic action. Figure 4 shows that, as in normal acini (Matthews & Cui, 1990), perifused single acinar cells also secrete increased amounts of amylase after photodynamic action. It can be concluded therefore that the photodynamic inhibition of amylase secretion by SALPC is not attributable simply to the fact that AR4-2J cells do not form a complete acinus under culture conditions.

**Figure 4** Photodynamic action of SALPC on amylase release from perifused single acinar cell preparations. Cells were exposed to SALPC, 1 μM (○) from minutes 34 to 44 and illuminated for 10 min at 4,500 lux (horizontal bar). Control cells were not exposed to SALPC before illumination (●). When s.e. bars are not seen they lie within the symbols ($n = 4$).

**Figure 3** Photodynamic action of SALPC on amylase release from AR4-2J cells: modulation by dexamethasone. Cells, pretreated with dexamethasone 100 nM for 43 h, were exposed to SALPC, 1 μM (○) from the minutes 34 to 44 and illuminated for 10 min at 4,500 lux (horizontal bar). Control cells (●) were exposed to light but not to SALPC. The inhibition of amylase release was significant ($P<0.05$; *) from minutes 67 to 71 ($n = 3$).

### *Photodynamic action of SALPC: permeabilisation of the plasma membrane*

Since the photodynamic action of SALPC in AR4-2J cells is of a completely different pattern from that in normal acinar cells isolated from the rat pancreas, it was necessary to determine whether the plasma membrane of AR4-2J cells was, like that of normal cells, permeabilised by photodynamic action. In these experiments, the leakage of LDH from the perifused cells during photodynamic action was used to assess the time course and extent of the permeabilisation process.

Illustrated in Figure 5 is the simultaneous release of amylase and LDH from the same population of perifused AR4-2J cells during the photodynamic action of SALPC. Photon activation of SALPC caused an immediate decrease (inhibition) of amylase secretion and this inhibition persisted until the end of the experiment. In contrast, photodynamic action caused an increase in LDH leakage from the same population of cells and the increase was significantly different from controls ($P<0.05$) from minute 75 until the end of the experiment. The conclusion therefore is that the plasma membrane of AR4-2J cells was permeabilised by photodynamic action (SALPC 1 μM, 4,500 lux) and that molecules as large as LDH (130 kDa) could then diffuse from the cell into the bathing medium. The time course of LDH efflux was obviously delayed compared to the more rapid inhibition of amylase release.

### *Effect of digitonin on amylase release*

In view of the fact that the previous experiments have demonstrated that one aspect of the photodynamic action of SALPC is to permeabilise the cell membrane it was important to compare this effect with that of other chemical agents known to permeabilise the cell membrane directly. Digitonin was chosen for this purpose and its effects on amylase release are shown in Figure 6. Exposure to digitonin (10 μg $ml^{-1}$) caused an immediate increase in amylase release both from isolated acini and from cultured AR4-2J cells. The increase in amylase release from perifused AR4-2J cells was statistically significant ($P<0.05$) from controls from minutes 61 to 71, i.e. the rate of amylase release returned to control levels after digitonin withdrawal from the perifusion buffer. On the other hand, the increase in amylase release from perifused acini was significant from minutes 61 to 75, i.e. the rate of amylase release did not return immediately to control levels after digitonin withdrawal from the perifusion buffer.

**Figure 5** Photodynamic action of SALPC on amylase release (**a**, $n = 6$) and LDH efflux (**b**, $n = 6$) from the same population of perifused AR4-2J cells. Cells were exposed to SALPC, 1μM (□) from minutes 34 to 44 and illuminated for 10 min at 4,500 lux (horizontal bars). Control cells (●) were not exposed to SALPC before illumination. Amylase release was inhibited from minute 59 onwards ($P<0.05$; *) and LDH efflux was increased significantly from minute 75 onwards ($P<0.05$; *).

*Effect of A23187 on amylase release*

A further membrane active agent, but one with a different mechanism of action, i.e. the calcium ionophore, A23187, was also examined for its effects on amylase release. Once incorporated into the membrane, A23187 facilitates the transport of calcium down its concentration gradient into the cell (Dobler, 1981).

The effect of A23187 on amylase release is illustrated in Figure 7. Addition of A23187, 1 μM to the perifusion buffer caused an immediate increase in amylase release both from perifused AR4-2J cells and isolated pancreatic acini. The effect on AR4-2J cells was small and transient with a significant increase in amylase release at minute 61 only. In contrast, in perifused pancreatic acini, the rate of amylase release increased progressively with time after exposure to A23187, reaching a plateau 4 min after exposure to A23187 (at minute 65) and persisting at higher amylase output values until the end of the experiment.

*Photodynamic action of SALPC: ultrastructure of AR4-2J cells*

The fact that SALPC photodynamically inhibits amylase release from AR4-2J cells yet also permeabilises the cells and causes a loss of cytosolic LDH makes it important to establish whether these effects were accompanied by any major ultracytological changes. Figure 8a and b illustrates the ultrastructural characteristics of AR4-2J cells not subjected to photodynamic action but exposed only to light. There are many microvilli in the intercellular space, but there is no

**Figure 6** Effect of digitonin on amylase release from perifused AR4-2J cells (**a**) and from normal pancreatic acini (**b**). Cells were exposed to digitonin, 10 μg ml$^{-1}$ (○) for the time indicated by the horizontal bar. In control experiments (●) the cells were not exposed to digitonin but perifused with normal medium throughout ($n = 3$).

**Figure 7** Effect of A23187 on amylase release from perifused AR4-2J cells (**a**) and from normal pancreatic acini (**b**). Cells were exposed to A23187, 1 μM (○) for the time indicated by the horizontal bar. Control cells (●) were not exposed to A23187 ($n = 3$).

evidence of junctional complexes between cells. The cells have a large irregular nucleus, abundant mitochondria and free ribosomes, sparse endoplasmic reticulum and a few Golgi complexes but no zymogen granules. Figure 8c and d are of AR4-2J cells after photodynamic action. No apparent change in cell structure was observed. Figure 8d shows the

**Figure 8** Ultrastructure of AR4-2J cells. Sections **a** and **b** are of AR4-2J cells exposed to light (4,500 lux, 10 min) in the absence of SALPC; **c** and **d** are of AR4-2J cells after photodynamic action (SALPC, 1 μM, 4,500 lux, 10 min). Note that **b** and **d** are at higher magnification than **a** and **c**. Calibration bar: 1 μm.

cells at higher magnification: the sparse endoplasmic reticulum should again be noted together with the absence of electron-dense zymogen granules.

### Discussion

We have shown unequivocally that amylase secretion is inhibited by the photodynamic action of SALPC on AR4-2J cells. This is in sharp contrast to the stimulatory photodynamic effects of SALPC on amylase release from normal acinar cells (see present experiments and Matthews & Cui, 1989*a*, 1990). Our results also establish that the configuration of the acinus is not an important factor governing the stimulatory effect of photodynamic action in normal acinar cells. The inhibitory effect in AR4-2J cells must therefore be due to differences at the cellular level other than those simply of microanatomical arrangement, although of course this does not rule out a role for the mesenchymal matrix, including the vasculature, in contributing to the action of photodynamic drugs on tumours *in vivo*.

One major distinction between normal and AR4-2J cells is that the basal percentage amylase secretion in AR4-2J cells is about four times greater than that in normal acinar cells, although the former contain much less amylase in total. It has been reported recently (Swarovsky *et al.*, 1988) that AR4-2J cells release their secretory proteins, those of both basal and secretagogue-induced secretion, exclusively via a constitutive process of secretion, which differs from the regulated pathway for exocytotic secretion in normal cells. In regulated secretion from normal cells, the secretory products are manufactured in the Golgi and concentrated and stored in electron-dense secretory granules, which have a half-life of $>10$ h; they are therefore readily observed by electron microscopy. Regulated secretion can be triggered by external stimuli such as neurotransmitters and hormones via exocytosis of the secretory granules; indeed the exocrine pancreas is the archetypical example for this kind of secretion (Palade, 1975). In constitutive secretion, the secretory products are not concentrated or stored in secretory granules, but rather the products are secreted promptly as soon as they are synthesised. The secretory material is rapidly transported in small vesicles from the Golgi complex and gains the exterior by a fission-fusion process at the plasma membrane. These transport vesicles have half-life of only some 10 min and are not electron-dense (Geuze & Slot, 1980; Kelly, 1985). In AR4-2J cells, such transport vesicles are therefore likely to be responsible for basal amylase secretion via the constitutive pathway with freshly synthesised amylase largely confined to these small transport vesicles. A further possibility, that of the existence of a large pool of amylase free in the cytosol is effectively eliminated, since amylase (50 kDa) was not released from the cytoplasm when a cytosolic molecule as large as LDH (130 kDa) was released after membrane permeabilisation (Figure 5).

Experiments on the possible contribution of a more restricted store of amylase were carried out with A23187, the calcium ionophore. These experiments demonstrate that, despite its continued presence in the perifusion medium, A23187 induced only a small, transient increase of amylase release. Since A23187 stimulated amylase secretion is dependent on a selective transmembrane uptake of $Ca^{2+}$ and not on secretagogue membrane receptors, which are effectively bypassed, the transitory nature of the response is not due to receptor desensitization, as could be the case with agonists such as substance P. Considering the lack of zymogen granules in the AR4-2J cell, the effect may therefore again, as with substance P, be due to rapid depletion of a small amylase store. The amylase store could take the form of nascent storage vesicles less mature than zymogen granules, but more mature than transport vesicles, and not readily identifiable as dense vesicles by electron microscopy.

Functional receptors for substance P (Womack *et al.*, 1985), CCK (Logsdon, 1986; Scemama *et al.*, 1988), VIP and somatostatin (Viguerie *et al.*, 1987, 1988) have been reported to exist in AR4-2J cells. Stimulation of substance P receptors leads to an increase in amylase secretion, as confirmed in this work. On the other hand, CCK is reportedly able to increase or inhibit amylase secretion in AR4-2J cells (Scemama *et al.*, 1988). Somatostatin receptors were found to be coupled to a $N_i$ ($G_i$) protein and therefore on stimulation inhibit the VIP response (Viguerie *et al.*, 1987, 1988). Unless the relative density of these various receptors is established, it is not clear whether simultaneous stimulation of all receptors or G-proteins during the photodynamic action of SALPC would be expected to result in the inhibition or, as in normal cells, stimulation of amylase release (Matthews & Cui, 1989*a*, 1990). The receptor population and their associated transducer systems may therefore be an important determinant in the action of photodynamic drugs.

Another possibility is that inhibition of amylase secretion by photodynamic action reflects a more direct inhibition of constitutive secretion. Plasma membrane proteins are likely to play a vital role in the fission-fusion process between the transport vesicles and the plasma membrane; their oxidation by singlet oxygen in photodynamic action would then effectively block constitutive secretion. It is also possible that after dexamethasone treatment, the opposing effects of photodynamic inhibition of constitutive secretion and stimulation of a transcription-enhanced regulated secretion leads to the net result observed in Figure 3.

The continued inhibition of amylase secretion after photodynamic action (SALPC, 1 μM, light, 4,500 lux) is particularly intriguing. Experiments with LDH leakage clearly indicate that after photodynamic action, the plasma membrane of AR4-2J cells was permeabilised; yet calcium influx (the cells were permeabilised in solution containing $Ca^{2+}$, 2 mM) did not evoke amylase release; the calcium ionophore A23187 also had relatively little effect on AR4-2J cells. In contrast, direct membrane permeabilisation with digitonin elicits amylase release from both normal and AR4-2J cells. Based on the available evidence the conclusion must therefore be that, after photodynamic action, membrane proteins or lipids important for constitutive secretion are inactivated by the generation of singlet oxygen leading to the inhibition of amylase secretion. The molecular nature of the membrane components involved will be the subject of future investigation.

In conclusion, direct membrane permeabilisation of AR4-2J cells either with digitonin or with the calcium ionophore A23187 leads to amylase secretion due to calcium influx. On the other hand, the photodynamic action of SALPC on AR4-2J cells is to inactivate those membrane proteins or lipids that are important for the maintenance of constitutive secretion via the transport vesicle system. Constitutive secretion by exocytosis of zymogen granules after dexamethasone treatment may not involve these proteins and is, in consequence, less sensitive to photodynamic action; the overall result is therefore that after dexamethasone treatment photodynamic inhibition by SALPC is alleviated. Photodynamic inhibition of amylase secretion always precedes membrane permeabilisation, but the fact that subsequent membrane permeabilisation (and calcium influx) did not evoke amylase secretion confirms that any membrane proteins or lipids involved in constitutive secretion via the transport vesicle system are especially susceptible to photodynamic action. Finally, although the precise mechanisms remain to be fully resolved, this study has disclosed a major difference between the photodynamic action of SALPC on the secretory machinery of normal cells and those derived from a pancreatic carcinoma. Further investigations are therefore now urgently required to identify the molecular targets involved since with these observations as a starting point it may be possible to develop therapeutic agents with a selective photodynamic action on pancreatic tumour cells (Mang & Wieman, 1988).

We are grateful to Dr M.R. Hanley for providing the AR4-2J cells and for the use of tissue culture facilities. We also thank the British Council and St John's College, Cambridge, for financial support. Z.J.C. is a British Council Chinese Scholar.

### References

AMSTERDAM, A., SOLOMON, T.E. & JAMIESON, J.D. (1978). Sequential dissociation of the exocrine pancreas into lobules, acini, and individual cells. In *Methods in Cell Biology XX*, Prescott, D.M. (ed.) p. 361. Academic Press: New York.

BRADFORD, M.M. (1976). A rapid and sensitive method for the quantitation of microgram quantities of protein utilizing the principle of protein-dye binding. *Anal. Biochem.*, **72**, 248.

CESCA, M., BIRATH, K. & BROWN, B. (1969). A new and rapid method for the clinical determination of α-amylase activities in human serum and urine. Optimal conditions. *Clin. Chim. Acta.*, **26**, 437.

CHAN, W.-S., SVENSEN, R., PHILLIPS, D. & HART, I.R. (1986). Cell uptake, distribution and response to aluminium chlorosulphonated phthalocyanine, a potential anti-tumour photosensitizer. *Br. J. Cancer*, **53**, 255.

CUI, Z.J. & MATTHEWS, E.K. (1989). Amylase secretion from isolated rat pancreatic acini and cultured AR4-2J cells: effects of a photodynamic agent. *J. Physiol.*, **416**, 36P.

DOBLER, M. (1981). *Ionophores and Their Structures*. Wiley: New York.

ELEVITCH, F.R. & PHILLIPS, R.E. (1966). *Lactate Dehydrogenase in Serum*. G.K. Turner: Palo Alto, CA.

GEUZE, H.J. & SLOT, J.W. (1980). The subcellular localization of immunoglobulin in mouse plasma cells, as studied with immunoferritin cytochemistry on ultrathin frozen sections. *Am. J. Anat.*, **158**, 161.

JESSOP, N.W. & HAY, R.J. (1980). Characteristics of two pancreatic exocrine cell lines derived from transplantable tumours. *In Vitro*, **16**, 212.

KELLY, R.B. (1985). Pathways of protein secretion in eukaryotes. *Science*, **230**, 25.

LOGSDON, C.D. (1986). Glucocorticoids increase cholecystokinin receptors and amylase secretion in pancreatic acinar AR4-2J cells. *J. Biol. Chem.*, **261**, 2096.

LOGSDON, C.D., MOESSNER, J., WILLIAMS, J.A. & GOLDFINE, I.D. (1985). Glucocorticoids increase amylase mRNA levels, secretory organelles and secretion in pancreatic acinar AR4-2J cells. *J. Cell Biol.*, **100**, 1200.

LONGNECKER, D.S, LILJA, H.S., FRENCH, J., KUHLMANN, E. & NOLL, W. (1979). Transplantation of azaserine-induced carcinomas of pancreas in rats. *Cancer Lett.*, **7**, 197.

MANG, T.S. & WIEMAN, T.J. (1988). An investigation of photodynamic therapy in the treatment of pancreatic carcinoma: dihematoporphyrin ether uptake and photobleaching kinetics. *Proc. SPIE*, **847**, 116.

MANYAK, M.J., RUSSO, A., SMITH, P.D. & GLASTEIN, E. (1988). Photodynamic therapy. *J. Clin. Oncol.*, **6**, 380.

MATTHEWS, E.K. & CUI, Z.J. (1987). Photodynamic drug action on smooth muscle. *Proc. Xth Int. Congr. Pharmacol.*, p. 6.

MATTHEWS, E.K. & CUI, Z.J. (1989*a*). Photodynamic drug action on rat pancreatic acini. *Br. J. Pharmacol.*, **97**, 430P.

MATTHEWS, E.K. & CUI, Z.J. (1989*b*). Photodynamic action of rose bengal on isolated rat pancreatic acini: stimulation of amylase release. *FEBS Lett.*, **256**, 29.

MATTHEWS, E.K. & CUI, Z.J. (1990). Photodynamic action of sulphonated aluminium phthalocyanine (SALPC) on isolated rat pancreatic acini. *Biochem. Pharmacol.* (in the press).

MATTHEWS, E.K. & MESLER, D.E. (1984*a*). Photodynamic action of halogenated fluorescein derivatives on smooth muscle cells. *J. Gen. Physiol.*, **84**, 24a.

MATTHEWS, E.K. & MESLER, D.E. (1984*b*). Photodynamic effects of erythrosine on the smooth muscle cells of guinea-pig *Taenia coli*. *Br. J. Pharmacol.*, **83**, 555.

PALADE, G. (1975). Intracellular aspects of the process of protein synthesis (secretion). *Science*, **189**, 347.

ROGERS, J.R., HUGHES, R.G. & MATTHEWS, E.K. (1988). Cyclic GMP inhibits protein kinase C-mediated secretion in rat pancreatic acini. *J. Biol. Chem.*, **263**, 3713.

SCEMAMA, J.L., ROBBERECHT, P., WAELBROECK, M. & 4 others (1988). CCK and gastrin inhibit adenylate cyclase activity through a pertussis toxin-sensitive mechanism in the tumoural rat pancreatic acinar cell line AR4-2J. *FEBS Lett.*, **242**, 61.

SWAROVSKY, B., STEINHILBER, W., SCHEELE, G.A. & KERN, M.F. (1988). Coupled induction of exocrine proteins and intracellular compartments involved in the secretory pathway in AR4-2J cells by glucocorticoids. *Eur. J. Cell Biol.*, **47**, 101.

TRALAU, C.J., BARR, H., SANDEMAN, D.R. & 3 others (1987). Aluminium sulfonated phthalocyanine distribution in rodent tumours of the colon, brain and pancreas. *Photochem. Photobiol.*, **46**, 777.

VIGUERIE, N., ESTEVE, J., SUSIONI, C., LOGSDON, C.D., VAYSSE, N. & RIBET, A. (1987). Dexamethasone effects on somatostatin receptors in pancreatic acinar AR4-2J cells. *Biochem. Biophys. Res. Commun.*, **147**, 942.

VIGUERIE, N., TAHIRI-JOUTI, N., ESTEVE, J. & 6 others (1988). Functional somatostatin receptors on a rat pancreatic acinar cell line. *Am. J. Physiol.*, **255**, G113.

WEISHAUPT, K.R., GOMER, C.J. & DOUGHERTY, T.J. (1976). identification of singlet oxygen as the cytotoxic agent in photoactivation of a murine tumour. *Cancer Res.*, **36**, 2326.

WOMACK, M.D., HANLEY, M.R. & JESSELL, T.M. (1985). Functional substance P receptors on a rat pancreatic acinar cell line. *J. Neurosci.*, **5**, 3370.

YONUSCHOT, G., MATTHEWS, E.K., CORPS, A.N. & METCALFE, J.C. (1987). Permeabilization of thymocytes by photon activation of erythrosin. *FEBS Lett.*, **213**, 401.